# Percutaneous transhepatic biliary stent placement in the palliative management of malignant obstructive jaundice: initial experience in a tertiary center in Ghana

**DOI:** 10.11604/pamj.2020.37.96.20050

**Published:** 2020-09-27

**Authors:** Benjamin Dabo Sarkodie, Benard Ohene Botwe, Edmund Kwadwo Kwakye Brakohiapa

**Affiliations:** 1 Department of Radiology, School of Medicine and Dentistry, University of Ghana, Accra, Ghana,; 2 Department of Radiography, School of Biomedical and Allied Health Sciences, College of Health Sciences, University of Ghana, Accra, Ghana

**Keywords:** Percutaneous, palliative, obstructive jaundice, stent, Ghana

## Abstract

**Introduction:**

one of the mainstays of management of malignant biliary obstruction is the decompression of the biliary system and its associated obstructive symptoms. Non-surgical palliative treatment such as percutaneous transhepatic biliary stenting is desirable in many selected patients. However, this service is often not available in many resource-limited countries. We share our initial experience of percutaneous transhepatic biliary stenting for the management of malignant biliary obstruction in our first set of patients with surgically non resectable malignant biliary obstruction in Ghana.

**Methods:**

percutaneous transhepatic biliary stenting was performed on the first 23 consecutive patients at the Korle Bu Teaching Hospital. The procedure served as the first palliation for malignant obstruction through interventional radiology. Medical records as well as serum levels of total bilirubin (TBil), aspartate aminotransferase (AST), alanine aminotransferase (ALT) and alkaline phosphatase (ALP) were used to assess the efficiency of the intervention. Microsoft Excel 2010 was used to analysis the data.

**Results:**

most patients had resolution of jaundice with marked improvement in liver function and resolution of the itching associated with obstructive jaundice. During the follow-up of cases, one major complication of hemoperitoneum occurred requiring laparotomy. No other major complications such as bile leakage or death occurred. Four (4) patients had sepsis, which was managed.

**Conclusion:**

the introduction of the intervention in Ghana has proven to valuable for palliative drainage and relief of obstructive symptoms, hence contributing to better patient management. It is relatively safe with minor complications among Ghanaians with non-resectable obstructive symptoms.

## Introduction

Obstructive jaundice may be caused by benign or malignant disease processes. Malignant biliary tract obstruction is a common clinical entity [1]. Patients with tumors causing biliary tract obstruction are often asymptomatic until the diseases are significantly advanced [1]. The primary goal in the treatment of malignant obstruction of the biliary system is the relief of jaundice. Although operative biliary bypass is a reliable method of palliation, non-operative palliation may be desirable for selected patients [2]. Percutaneous transhepatic biliary drainage stent placement is intended for patients with non-operable lesions [3]. In such patients´ biliary drainage/stent plays an important role in treatment. Interventional drainage and stenting can normalize plasma bilirubin levels [4] and alleviate symptoms associated with biliary obstruction [5], leading to improvement in quality of life and thus optimizing the clinical state of patients to allow for resection or palliative radio or chemotherapy [6,7]. However, this important intervention is not readily available in the most resource poor countries because of the limited number of interventional radiologists. Ghana, with a population of about 25 million has now three interventional radiologists. Percutaneous transhepatic biliary stenting (PTBS) services have taken off not long ago. This paper is therefore directed at discussing the initial experience of PTBS performed in Ghana on 23 consecutive patients with irresectable malignant biliary obstruction. In this study, we evaluated the patient demographics, aetiology of obstructive jaundice, clinical status, changes in laboratory data and performance status, to assess the usefulness of percutaneous biliary stenting for the treatment of obstructive jaundice in Ghana.

## Methods

**Patients:** percutaneous biliary stenting was performed on 23 consecutive patients (17 women and 6 males) at the Korle Bu Teaching Hospital. In line with ethical considerations, patients´ consents were sought. All these patients were included in this study using purposive sampling technique after granting consent. All these patients did not have serious coagulation disorders or ascites. However, the causes of their obstructions were as follows: pancreatic carcinoma (n=5), metastases from a variety of primary sites (n=3), gallbladder carcinoma (n=2), cholangiocarcinoma (n=7) and undiagnosed (n=6). Thirteen of these patients underwent secondary stenting after initial PTBD while 10 patients underwent primary biliary stenting with self-expanding metallic stent (SEMS).

**Procedure:** the site and extent of obstruction were studied using abdominal ultrasound, enhanced computed tomography (CT) and/or magnetic resonance imaging (MRI) /magnetic resonance cholangiopancreatography (MRCP) pre-stenting. In each case, the accessing trajectory and puncture site were determined under ultrasonography. With the patient in a supine position on the examination table, ultrasonography (US) to show intrahepatic biliary ductal dilatation was performed and routine sterile preparation was also done. After administration of local anaesthesia/peripheral duct was punctured successfully by a 22G Chiba needle under US guidance. A peripheral bile duct was punctured successfully by a 22G Chiba needle under US guidance. A limited cholangiography was made to delineate the extent of intrahepatic biliary dilatation. Then a 0.018” steerable mandril wire was passed through the Chiba needle coaxially to the hilum or just above the level of obstruction. The puncture tract was serially dilated by a 3-piece aprima (cook, Indiana), over the steerable guide wire. Tract dilatation was done up to 6F and a 6F outer piece/sheath of the aprima was inserted over the guide wire. A 0.035” hydrophilic guide wire was used to cross the obstruction under the assistant of 4F Berestein. Once the obstruction was crossed, cholangiography was performed again in order to determine the nature and length of obstruction. The guide wire was then advanced into the duodenum and exchanged with a stiff 0.035” Amplatz guide wire.

An appropriate size of the stent was advanced across the stenosis over the Amplatz guide wire under fluoroscopy. Then the self-expandable metallic stent was deployed under fluoroscopy. In 2 of our patients, the guide wire could not be manipulated after the obstruction and therefore, external drainage catheters were inserted along the guide wire. After 3-5 days drainage, re-attempt was made again and the stents were deployed. Post stenting antibiotics were used routinely. In all, 24 stents were implanted in the 23 patients. Twenty patients with common bile duct obstruction had a single stent inserted across the ampulla of Vater (transampulary), 2 were placed above the ampulla (supra ampullary). In 1 case, 2 stents were implanted in the left and right hepatic ducts up to the common bile duct in a Y-fashion, as there was non-communicating hilar obstruction. Symptoms of patients were recorded before and one week after the procedure to test for the effectiveness of the procedure statically. In addition, mean serum levels were recorded before and one month after the procedure. Microsoft Excel 2010 was used to analysis the data. The usefulness of the procedure was evaluated with Chi-square.

## Results

A total of 23 patients (17 women and 6 males, mean age 57 years, age range, 42-79 years) (Table 1) who had inoperable malignant biliary obstruction and underwent percutaneous biliary stenting (first beneficiaries of the intervention) at the Korle-Bu Teaching Hospital participated in the study. Causes of biliary obstructions were as follows: pancreatic carcinoma (n=5), metastases from a variety of primary sites (n=3), gallbladder carcinoma (n=2), cholangiocarcinoma (n=7) and undiagnosed (n=6). The causes of obstructive jaundice were studied using abdominal computed tomography (CT), abdominal ultrasonography, image guided biopsy and transluminal brush biopsy. The indication for stenting was the presence of symptoms caused by obstructive jaundice, which were surgically unresectable or where patients were not fit for anaesthesia. After successful biliary stenting procedures, 10 patients received chemotherapy and the rest received best supportive care. The usefulness of the percutaneous biliary stenting procedures based on symptoms one week after and changes in laboratory data a month on, (Table 2) are presented in Figure 1. Most 16 (70%) of these patients could not afford the full cost of the procedure and therefore the procedures were heavily subsidised while others (10) underwent primary stenting. After stenting, most symptoms resulting from obstructive jaundice were relieved within one week, but for generalized malaise in three patients (Table 2). The differences in mean serum levels of total bilirubin (TBil), aspartate aminotransferase (AST), alanine aminotransferase (ALT) and alkaline phosphatase (ALP) after one month (Figure 1) were significant at (χ^2^=40.23 p=0.001), (χ^2^=37.12 p=0.005), (χ^2^=13.6 p=0.003) and (χ^2^=18.34 p=0.001) respectively.

**Table 1:** patient's characteristics

Characteristics		Frequency	%
**Sex (M/F)**		**6/17**	**21/79**
**Age**	**Mean (57 years)**		
	30-40 years	1	4.3
	40-50 years	3	13.0
	51-60 years	6	26.1
	61-70 years	11	47.8
	71+ years	2	8.8
Causes of obstruction	Cholangiocarcinoma	7	30.0
	Pancreatic cancer	5	22.0
	Gallbladder carcinoma	2	8.8
	Metastatic disease	3	13.0
	Undiagnosed/other	6	26.1
Level of obstruction	Hilum	1	4.3
	Common bile duct	22	95.7

**Table 2:** changes in symptoms 1-week post stenting

Initial symptom	Before the intervention	One week after the intervention			
	# of persons with symptoms	# of persons with disappeared symptoms	# of persons with decreased symptoms	# of persons with no change in symptoms	# of persons with increased symptoms
Skin itching	19	19	0	0	0
Generalized malaise	15	10	2	3	0
Nausea	6	5	1	0	0
Abdominal fullness	4	0	4	0	0
Jaundice	23	5	15	0	0

# = number

**Figure 1:**
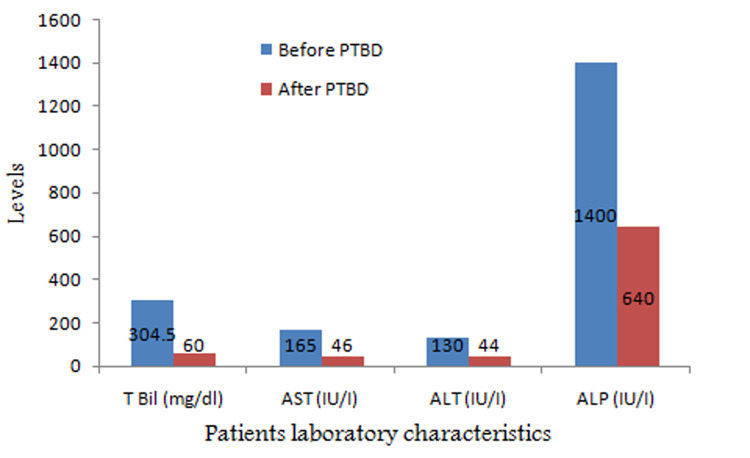
changes in mean laboratory data before and after PTBS

## Discussion

PTBD biliary stenting is a palliative intervention that prolongs life of the patient with inoperable obstructive malignant jaundice [8-11]. However, in developing countries where there is a lack of interventional radiologists and ERCP skills, this service is often not available. Percutaneous biliary stenting done by a skilled Interventional radiologist has minimal complications, however, Ghana until recently had no personnel to support this service. Therefore, our initial experience in terms of availability of dedicated equipment and materials, patients´ understanding of the procedure as well as staff support was limited. Lack of funds was a major challenge as most of the patients could not afford the cost of stents and other consumables. In all, 23 patients were managed with PTBS. Causes of biliary obstruction were as follows: gallbladder carcinoma, pancreatic carcinoma, metastases from a variety of primary sites, cholangiocarcinoma and undiagnosed. These were causing skin itching, generalized malaise, nausea, abdominal fullness and jaundice. The causes of obstructive jaundice and bile duct strictures were investigated using abdominal computed tomography, abdominal ultrasonography and image guided biopsy prior to the procedure.

In our experience, one major complication of hemoperitoneum occurred requiring laparotomy as embolization coils were not available for endovascular treatment. Otherwise, no other major complications such as bile leakage or death occurred. Four (4) patients had sepsis and they were managed with antibiotics. Some studies have found comparable rates of complications during endoscopic and percutaneous biliary stenting [8,12]. Interestingly, most of the symptoms disappeared or decreased in the patients (Table 2), as relieving bile duct obstruction with subsequent restoration of bile flow into the duodenum led to an immediate improvement in the patient´s general condition. The drop in the mean serum levels of total bilirubin (TBil), aspartate aminotransferase (AST), alanine aminotransferase (ALT) and alkaline phosphatase (ALP) after one month (Figure 1) was statistically significant at p=0.001, p=0.005, p=0.003 and p=0.001 respectively, emphasising the usefulness of the intervention in Ghana. Self-expanding metallic prostheses retain patency for a long time and are by many considered management of choice [12-14], however, in developing countries such as Ghana, the “high” cost of stents prevents many people from having the procedure.

Most of these patients could not afford the full cost of the procedure and therefore the procedures had to be heavily subsidized while others (10) underwent primary stenting to reduce their fees. The government and other organizations could help alleviate the financial challenges hindering this important intervention in this resource poor environment. Moreover, the aim of palliative treatment for terminal stage conditions as discussed is to enable patients to return home and avoid prolonged stay in the medical centres [10]. Therefore, with good cooperation with the patients and the families PTBS offers relief and improves clinical outcomes in patients with malignant biliary obstruction.

## Conclusion

We conclude that PTBS is useful for relief of symptoms, caused by obstructive jaundice among our first set of patients in Ghana. PTBS offers a safe and effective method in providing palliative treatment for patients with biliary obstruction. It eliminates the need for open surgery and avoids the problems associated with anaesthesia. Many patients felt better after the procedure and also helped to integrate them better into their families. However, in our initial experience in the developing country of Ghana, availability of dedicated equipment and materials, patients´ appreciation of the procedure as well as staff support was limited. Lack of funds was a major challenge as most of the patients also could not afford the cost of stents and the procedure wires. Therefore, financial support is needed to promote the intervention in as Ghana.

### What is known about this topic

- One of the mainstays of management of malignant biliary obstruction is the decompression of the biliary system and its associated obstructive symptoms;
- Non-surgical palliative treatment such as percutaneous transhepatic biliary stenting (PTBS) is desirable in many selected patients. However, this service is often not available in many resource-limited countries.

### What this study adds

- Our initial experiences of PTBS in Ghana have proven valuable for palliative drainage and relief of obstructive symptoms, hence contributing to better patient management;
- PTBS is relatively safe with minor complications among the Ghanaians (used in the study) with non-resectable obstructive symptoms.
